# Psychological abuse and its influencing factors among home-dwelling older people in Northern China: a cross-sectional survey

**DOI:** 10.3389/fmed.2024.1492826

**Published:** 2025-01-15

**Authors:** Xiaomeng Wu, Li Pei, Yu Wang, Lanrui Zhang, Dongqing Zhao, Haoying Dou

**Affiliations:** ^1^Tianjin University of Traditional Chinese Medicine, Tianjin, China; ^2^School of Nursing, Rongjun Hospital, Lvliang, Shanxi, China

**Keywords:** abuse, psychological abuse, older people, activity of daily living, family functioning

## Abstract

**Background:**

As the population ages, the subject of elder abuse has become more prominent, with psychological abuse of older people being particularly prevalent. This leads to a higher incidence of anxiety, depression, and other psychological problems among older people, reducing their quality of life, and even jeopardizing their safety.

**Methods:**

A cross-sectional questionnaire survey was conducted to investigate psychological abuse among home-dwelling older people in Northern China and its influencing factors. Participants were surveyed using a demographic questionnaire, activities of daily living (ADL), family adaptability and cohesion evaluation scale (FACES), and elder psychological abuse scale. The factors impacting psychological abuse were examined using binary logistic regression.

**Results:**

A total of 465 home-dwelling older people participated in the study, with an effective response rate of 97.89%. The median item score of psychological abuse was 20 (IQR: 18–22), activities of daily living was 15 (IQR: 14–17), and family adaptability and cohesion was 96 (IQR: 86–105). Binary logistic regression analysis indicated that the factors affecting psychological abuse among home-dwelling older people were residence in rural areas (OR = 3.487, 95% CI = 2.121–5.732), monthly income ≥ 3,501 (OR = 0.342, 95% CI = 0.119–0.987), had chronic diseases (OR = 2.202, 95% CI = 1.356–3.576), and FACES (OR = 0.955, 95% CI = 0.936–0.974) were the factors that influenced the prevalence of psychological abuse.

**Conclusion:**

In Northern China, the level of psychological abuse among older people is low. It is associated with residence, monthly income, chronic diseases, and family adaptability and cohesion. Further studies are required to understand the underlying causes and develop effective interventions to reduce psychological abuse and ensure a comfortable old life for older Chinese people.

## Introduction

China’s National Statistics show that by 2023, there will be 300 million people over the age of 60, or 21.1% of the overall population (1). Currently, the majority of older individuals in China prefer to live at home, however, they may be vulnerable to abuse from family members, friends, or caregivers. Elder abuse is a serious public health and social issue globally. The World Health Organization (WHO) categorizes elder abuse into 5 types, including physical, psychological, financial, and sexual abuse, and neglect (2). Psychological abuse is the most prevalent type of abuse, and it refers to behavior meant to inflict mental agony or harm on older people (3).

Psychological abuse of older people is widespread in both developed and developing countries. A systematic review of 28 countries worldwide found that the prevalence of elder abuse in community settings was 15.7%, with psychological abuse being the most common type (11.6%) (4). According to data on the prevalence of elder abuse in urban centers of seven European countries, 19.4% of older people were exposed to psychological abuse and this occurred more often in Sweden (29.7%) and Germany (27.1%) (5). A survey of Australian family caregivers and older people discovered that 40% had experienced abuse, with the highest rate of psychological abuse being 35% (6). American epidemiology data show that 4.6% of the 5,777 participants had experienced psychological abuse, and 11.4% of the participants had suffered at least one type of abuse in the previous year (7). The prevalence of psychological abuse in Japan was 11.12% (8), and in South Korea was 11% (9). Research on psychological abuse in older people started relatively late in China, and the subsequent studies were poor. An investigation was carried out on 689 families of older people in Chenzhou City, China. The total incidence of elder abuse was 35.6%, with psychological abuse accounting for the largest percentage (19.9%) (10). In Anhui province, China, 9.1% of older people experienced psychological abuse (11).

Psychological abuse has the most immediate and profound impact on the mental health of older people, compared with the other three types of elder abuse. Most studies have indicated that older people who have been psychologically abused are prone to anxiety, low self-esteem, depression, and even suicidal ideation (9, 12–14). Furthermore, older people who have experienced long-term psychological abuse may feel worthless in society and turn away from their families and other social interactions, forcing them into mandatory social isolation (15).

The influencing factors that contribute to older people being vulnerable to psychological abuse can be broken down into two categories: the characteristics of the older people and their caregivers. Previous studies have found that household registration, gender, age, and education level all influence psychological abuse among older people (12, 16). Caregivers’ stress, emotional status, and family relationships were also associated with psychological abuse (16, 17). The cultural backgrounds and subjects of each study differed, as did the contributing factors linked to psychological abuse. Traditional Chinese culture, which differs greatly from Western religious culture, has had a profound impact on older Chinese people. They often support a “child-centered” living state, prioritizing their children’s wellbeing but ignoring their own (18, 19). With the emergence of the nuclear family, the phenomenon of “valuing the small over the old” in Chinese society has become increasingly severe (20). The younger relatives devote more time and energy to their children while neglecting to care for older people, which may increase the risk of psychological abuse. It is crucial to explore the situation in China because there is currently insufficient research on the influencing factors that contribute to psychological abuse among older people in the country.

Recently, the issue of elder abuse in China has gained significant attention from community workers, medical experts, and legal professionals in recent times (21). Enhancing the quality of life for older people who are approaching the end of their lives requires a thorough assessment and discussion of the situation surrounding home-dwelling elders. Activities of daily living, such as the ability to eat, dress, wash, and others, are crucial indicators of health status and quality of life (22). Older people increasingly lose contact with the outer world as their everyday activities become less meaningful due to their physical limitations. As a result, they may be unable to provide financial support for their families and society and may face discrimination and neglect from their spouse or younger relatives (9, 23). In addition, family caregivers who are caring for older people with limited activities of daily living frequently experience fatigue, boredom, or pain, which may lead to negative behavioral outcomes such as psychological abuse. The family function reflects family members’ abilities to solve problems, communicate, adapt, and cohere (24). Close family relationships can provide older people with emotional support, improve communication, and reduce negative feelings (25, 26). This may potentially serve as a protective factor against psychological abuse among older people.

However, the current situation of psychological abuse, activities of daily living, and family functioning among Chinese home-dwelling older people has not been investigated, limiting the potential to develop personalized interventions to prevent their psychological abuse. Therefore, the purpose of this study is to: (1) examine the level of psychological abuse, activities of daily living, and family functioning among home-dwelling older people in Northern China (including three cities: Dalian, Liaoning province; Shijiazhuang, Hebei Province; and Jining, Shandong province); (2) explore the relationship between psychological abuse, activities of daily living, and family functioning among home-dwelling older people; and (3) analyze the influencing factors of psychological abuse.

## Materials and methods

### Study design

A descriptive, cross-sectional study was performed using a questionnaire.

### Setting and participants

The study included a self-report assessment of psychological abuse among Chinese home-dwelling older people. The sample was composed of 465 older people. Participants were recruited from three cities in northern China: Dalian, Liaoning Province; Shijiazhuang, Hebei Province; and Jining, Shandong Province. There are similarities in the social and cultural backgrounds of the older in all three cities. The inclusion criteria were older people aged ≥ 60, who had chosen home care. This study excluded participants who had been diagnosed with a cognitive impairment or a linguistic disability.

### Data collection

Before the survey, all researchers received uniform training to ensure the consistency of study results. Two researchers, as a team, entered the homes of the older people after getting their informed consent to conduct the survey. The older people were informed of the purpose, significance, cooperation, and confidentiality of the study before beginning the survey. The questionnaire was filled in by themselves. However, for older people who did not fill in the questionnaire on their own due to physical limitations or low literacy, the researchers read the questionnaire content in a neutral tone and recorded their answers. If conditions permit, the older people should fill in the questionnaire in a quiet and private place to ensure the reliability of the survey content.

### Data instruments

#### Sociodemographic characteristic information

The sociodemographic information included 10 variables, including gender, residence, education level, marital status, age, gender, marital status, monthly income, number of children, living style, source of income, and whether had chronic diseases.

#### Elder psychological abuse scale in domestic setting

This scale was developed by Jie (27). The scale consisted of 16 items divided into 4 dimensions: disrespect (5 items), isolation (3 items), threat (3 items), and control (3 items). The scale has a Likert range of 1 to 5, and the scores for “never/not applicable, occasionally, sometimes, often, always” were 1, 2, 3, 4, and 5 points, respectively. Higher scores indicate greater levels of psychological abuse experienced by older people. In this study, Cronbach’s α of this scale was 0.76.

#### Activities of daily living (ADL)

Lawton and Brody (28) devised the scale of ADL, which consists of two parts, namely the Physical Self-Maintenance Scale (PSMS) and instrumental activities of daily living (IADL). The PSMS consists of 6 items that focus on essential living activities such as dressing, eating, and maintaining personal cleanliness, as well as physical activities including sitting, standing, and walking. The IADL consists of 8 items that emphasize higher-level abilities in which people use instruments to live independently, such as housework, cooking, shopping, cycling, driving, and managing personal affairs. Each response was divided into four levels: 1 meant “No, I don’t have any difficulty,” 2 meant “I have difficulty but still can do it,” 3 meant “Yes, I have difficulty and need help,” and 4 meant “I cannot do it.” A score greater than 14 was defined as ADL impairment, with higher scores indicating more impaired activities of daily living. The scale was reliable and valid, with a Cronbach’s α score of 0.93.

#### Family adaptability and cohesion evaluation scale (FACES)

FACES is a commonly used family assessment tool. It was first proposed by Olson et al. (24). Later, Zhang et al. (29) translated it into Chinese for our country’s family setting, resulting in high reliability and validity. The scale was divided into two dimensions: Cohesion, the emotional connection between family members (16 items); Adaptability, the family system’s ability to cope with challenges resulting from family circumstances and different stages of family growth (14 items). Each item is scored on a 5-point Likert scale (from 1 = never to 5 = always), with higher scores indicating better family functioning. The survey yielded a Cronbach’s α value of 0.90.

### Data analysis

The Shapiro–Wilk test was used to verify if the numerical variables followed a normal distribution. The results showed that the data were not normally distributed. Consequently, frequencies and percentages were employed to describe categorical variables, whereas median and interquartile range (IQR) were utilized for continuous variables. A non-parametric test was employed to investigate significant variations in psychological abuse among participants with different characteristics. Spearman’s test was used to investigate the correlation between PSMS, IADL, FACES, and psychological abuse. Since the psychological abuse data were not normally distributed, the related factors of psychological abuse were estimated using binary logistic regression analysis. Statistical analyses were carried out using SPSS for Windows (version 27.0), with a two-tailed probability value of < 0.05 deemed statistically significant.

## Results

### Characteristics of the participants

A total of 475 questionnaires were distributed, and 465 valid surveys were included, resulting in a valid response rate of 97.89%. There were 120 cases from Jining City, 120 cases from Shijiazhuang City, and 225 cases from Dalian City, respectively. Among 465 older people, the age ranged from 60 to 93 years old, with an average age of (70.87 ± 6.71) years old. Males accounted for 49.9% and females accounted for 50.1%. Additional demographic data is displayed in Table 1.

**TABLE 1:** Characteristics of participants and univariate analysis of psychological abuse scores (*n* = 465).

Variables	*n* (%)	Median (IQR)	Z/H	*p*-value	Bonferroni test
Gender			−1.77	0.077	
Male	232 (49.9)	20 (18–21)			
Female	233 (50.1)	20 (18–22)			
Residence			−8.01	< 0.001	
Rural areas	312 (67.1)	20 (19–23)			
Cities and towns	153 (32.9)	19 (17–20)			
Education level			25.87	< 0.001	e < a, b, c; d < a, b, c
Illiteracy^a^	145 (31.2)	20 (19–22)			
Less primary school^b^	210 (45.2)	20 (19–22)			
Primary school^c^	83 (17.8)	20 (18–21)			
Secondary school^d^	23 (4.9)	18 (17–19)			
University or above^e^	4 (0.8)	17 (16.25–17.75)			
Marital status			20.83	< 0.001	a < b, a < d
Married^a^	368 (79.1)	20 (18–21)			
Divorced^b^	3 (0.6)	20 (19–21)			
Widowed^c^	87 (18.7)	21 (19–23.5)			
Remarried^d^	7 (1.5)	23 (21–27)			
Age			16.38	< 0.001	a, b < c
60–69 years old^a^	212 (45.6)	20 (18–21)			
70–79 years old^b^	201 (43.2)	20 (18–21)			
≥ 80 years old^c^	52 (11.2)	22.5 (19.25–25.75)			
Monthly income (RMB)			32.73	< 0.001	b, c, d, e < a; e < c
≤ 800^a^	176 (37.8)	20.5 (19–23)			
801–1,500^b^	125 (26.9)	20 (18–22)			
1,501–2,500^c^	83 (17.8)	20 (19–21)			
2,501–3,500^d^	45 (9.7)	19 (18–20.5)			
≥ 3,501^e^	36 (7.7)	19 (18–20)			
Number of children			4.76	0.190	
1	35 (7.5)	20 (18–21)			
2	186 (40)	20 (18–21)			
3	134 (28.8)	20 (18–22)			
≥ 4	110 (23.7)	20 (18–23.25)			
Living style			27.32	< 0.001	d < a, b, c; b < a, c
Live alone^a^	41 (8.8)	21 (20–23)			
Live with spouse^b^	225 (48.4)	20 (18–22)			
Live with children^c^	70 (15.1)	20.5 (19–24)			
Live with spouse and children^d^	129 (27.7)	19 (18–20.5)			
Source of income			28.11	< 0.001	b < a, e, f; a, d, e < f
Own salary income^a^	65 (14)	20 (18–21.5)			
Pension^b^	106 (22.8)	19 (18–21)			
Commercial insurance^c^	6 (1.3)	21 (18–21.5)			
Support by spouse^d^	45 (9.7)	20 (19–21)			
Provision of children^e^	160 (34.4)	20 (18–22)			
Government subsidies^f^	83 (17.8)	21 (20–24)			
Whether had chronic diseases			−3.64	< 0.001	
Yes	287 (61.7)	20 (19–22)			
No	178 (38.3)	19 (18–21)			

### Characteristics of the elder psychological abuse scale for home-dwelling older people scores

The median score on the psychological abuse scale for older people was 20 (IQR:18–22), with a total score ranging from 16 to 40 (Table 2).

**TABLE 2:** Characteristics of the scores on scales (*n* = 465).

Variables	Median (IQR)	Min–max	Item median (IQR)
Total score of psychological abuse	20 (18–22)	16–40	1.25 (1.13–1.38)
ADL	15 (14–17)	14–54	1.07 (1.00–1.21)
PSMS	6 (6–6)	6–22	1.00 (1.00–1.00)
IADL	9 (8–11)	8–32	1.13 (1.00–1.38)
FACES	96 (86–105)	50–140	3.20 (2.87–3.50)

ADL, activities of daily living; FACES, family adaptability and cohesion evaluation scale.

### Univariate analysis of psychological abuse scores

Univariate analysis revealed that the level of psychological abuse of the older people was significantly different in terms of residence, education level, marital status, age, monthly income, living style, source of income, and whether they had chronic diseases (*p* < 0.05). However, there was no difference in gender and number of children (*p* > 0.05). Subsequent *post hoc* comparisons are shown in Table 1.

### ADL, FACES scores and correlation with psychological abuse

The median score on the ADL of home-dwelling older people was 15 (IQR: 14–17), PSMS was 6 (IQR: 6–6), and IADL was 9 (IQR: 8–11). The median score on the FACES of home-dwelling older people was 96 (IQR: 86–105) (Table 2). ADL had a positive correlation with total psychological abuse scores for home-dwelling older people (rs = 0.222, *p* < 0.001). PSMS and IADL both had a positive correlation with total psychological abuse scores (rs = 0.199, *p* < 0.001, rs = 0.221, *p* < 0.001). FACES had a negative correlation with total psychological abuse scores (rs = −0.373, *p* < 0.001). This indicated that older people with higher PSMS and IADL impairment, and better family function were more likely to suffer psychological abuse. Additionally, a marginally positive correlation was discovered between the ADL and FACES scores (rs = −0.111, *p* < 0.001). The results indicated that the better the family function of the older, the lower the ADL impairment rate (Table 3).

**TABLE 3:** ADL, PAMS, IADL and FACES correlation with psychological abuse (*n* = 465).

	1	2	3	4	5
1. .ADL	1				
2. .PSMS	0.633**	1			
3. .IADL	0.995**	0.580**	1		
4. .FACES	−0.111*	−0.129**	−0.108*	1	
5. .Psychological abuse	0.222**	0.199**	0.221**	−0.373**	1

**p < 0.01, *p < 0.05.

### Binary logistic regression analysis of factors influencing psychological abuse among home-dwelling older people

Since the data on psychological abuse in this study did not conform to the normal distribution, the data were dichotomized according to the median of psychological abuse, and then binary logistic regression analysis was conducted. Univariate statistically significant independent variables as well as ADL, PAMS, IADL and FACES were entered into the binary logistic regression. The associations between the various variables were expressed as unstandardized Betas and their standard errors or Odds ratios (OR) and CI 95%. The results showed that residence in rural areas (OR = 3.487, 95% CI = 2.121–5.732), monthly income ≥ 3,501 (OR = 0.342, 95% CI = 0.119–0.987), had chronic diseases (OR = 2.202, 95% CI = 1.356–3.576), and FACES (OR = 0.955, 95% CI = 0.936–0.974) were the factors that influenced the psychological abuse (Table 4).

**TABLE 4:** Binary Logistic regression analysis of the factors influencing psychological abuse (*n* = 465).

Variables	Comparison Group	Reference group	B	SE	Waldχ^2^	*P*	OR	95% CI
Constant			3.941	1.342	8.618	< 0.001	51.469	
**Residence**								
	Cities and towns	Rural areas	1.249	0.254	24.255	< 0.001	3.487	2.121–5.732
Education level								
	Less primary school	Illiteracy	0.510	0.277	3.395	0.065	1.665	0.968–2.864
	Primary school		0.528	0.367	2.064	0.151	1.695	0.825–3.481
	Secondary school		−0.793	0.633	1.571	0.210	0.452	0.131–1.564
	University or above		−38.118	21,657.05	0	0.999	0	0
**Marital status**								
	Divorced	Married	19.821	14,612.983	0	0.999	405,772,250.3	0
	Widowed		0.205	0.549	0.139	0.709	1.227	0.418–3.60
	Remarried		−0.523	0.908	0.332	0.564	0.593	0.100–3.513
**Age**								
	70–79 years old	60–69 years old	0.168	0.267	0.394	0.530	1.183	0.700–1.997
	≥ 80 years old		0.552	0.520	1.126	0.289	1.736	0.627–4.809
**Monthly income (RMB)**								
	801–1,500	≤ 800	−0.453	0.337	1.811	0.178	0.636	0.328–1.23
	1,501–2,500		−0.101	0.408	0.061	0.805	0.904	0.406–2.012
	2,501–3,500		−0.978	0.498	3.857	0.050	0.376	0.142–0.998
	≥ 3,501		−1.072	0.54	3.937	0.047	0.342	0.119–0.987
**Living style**								
	Live with spouse	Live alone	−0.394	0.706	0.311	0.577	0.675	0.169–2.689
	Live with children		−0.498	0.579	0.738	0.390	0.608	0.195–1.892
	Live with spouse and children		−0.908	0.714	1.617	0.204	0.403	0.099–1.635
**Source of income**								
	Pension	Own salary income	−0.410	0.401	1.048	0.306	0.664	0.303–1.455
	Commercial insurance		−0.276	1.036	0.071	0.790	0.759	0.100–5.775
	Support by spouse		−0.140	0.464	0.091	0.763	0.869	0.350–2.158
	Provision of children		−0.721	0.410	3.098	0.078	0.486	0.218–1.085
	Government subsidies		−1.036	0.563	3.386	0.066	0.355	0.118–1.070
**Whether had chronic diseases**								
	No	Yes	0.789	0.247	10.187	0.001	2.202	1.356–3.576
PSMS			0.034	0.115	0.088	0.766	1.035	0.826–1.297
IADL			0.023	0.047	0.232	0.630	1.023	0.932–1.122
FACES			−0.046	0.010	21.64	0.003	0.955	0.936–0.974

SE, standard error; OR, odds ratio; CI, confidence interval.

## Discussion

This study is devoted to analyzing psychological abuse because there is comparatively little research in comparison to general abuse studies. The median total score of psychological abuse among home-dwelling older people was 21 (20,24), indicating that they were at a low level of psychological abuse, which was consistent with a previous study (27). This might profit from the continual improvement of China’s pension service system in recent years, including the senior subsidy system, medical insurance, social welfare, and so on. Older people’s quality of life can be greatly enhanced by this intervention, which also has the potential to reduce the risk of psychological abuse. Additionally, the older in China, who are greatly impacted by traditional culture, think that “Domestic shame should not be published” (30). They might conceal some “ugly” phenomena, which would lead to biased reporting. A study carried out by Wang (31). has shown that the prevalence of psychological abuse among older residents is higher than that reported by the Taiwanese government. As a result, psychological abuse is frequently kept as a family secret and further research is needed into the sensitivity and concealment. Beyond this scale for psychological abuse, more thorough assessments of older people are required in the future.

In the present study, a positive connection was found between the scores of PSMS, IADL, and the score of psychological abuse. Stated differently, when older people are severely impaired in physical maintenance or instrumental activities of daily living, their level of psychological abuse increases. It is consistent with the findings of Burnes et al. (32), Muldoon et al. (33), and Sooryanarayana et al. (34), who reported that older people with impairments in daily activities were more likely to suffer abuse, violent or destructive behaviors. On the one hand, as PSMA deteriorates among home-dwelling older people, they rely on spouses and younger relatives to assist them with essential living activities. In addition to increasing the strain on caregivers, this can cause depression, loss, diminished self-worth, low self-esteem, and other negative emotions among older people. Consequently, it raises the risk factors for psychological abuse. On the other hand, when the IADL level of older people decreases, their interaction with the outside world reduces, resulting in a lack of resources and value exchange, which cannot benefit the family. Then there is discrimination, neglect, and even psychological abuse by their spouses or relatives. This is just like the “social exchange theory” proposed by the American sociologist Homans, which states that through the exchange of resources with others to meet their own needs, emotional needs can also be exchanged (33). In addition, long-term psychological abuse by caregivers also impairs the PSMS and IADL ability. The interaction between the two creates a vicious cycle that lowers daily living activities and raises the risk of psychological abuse, which in turn causes older people’s health to deteriorate (35, 36). Therefore, community workers should periodically evaluate old people’s activities of daily living so as to strengthen monitoring and early warning.

The analysis of the association between FACES and psychological abuse scores showed significant differences. FACES is one of the key components of family function that can improve the emotional bond between family members and lessen older people’s cognitive impairment (37). In this study, older people with poor family functioning are more prone to experience psychological abuse. This is consistent with Li et al.’s (38) findings about the relationship between family type and abuse. It discovered that Chinese-American older people with unobligated, ambivalent, separated, and commanding conflicted family types were more likely to encounter elder abuse. Based on a power-oriented communication model, Lin and Giles (39) discovered that dysfunctional communication between the caregivers and care receivers may increase the chance of elder abuse. Therefore, there is a strong correlation between FACES and psychological abuse, and subsequent studies cannot overlook the function of family on elder abuse.

In addition, FACES was found to be a factor influencing psychological abuse FACES (OR = 0.955, 95% CI = 0.936–0.974), and good family function is a protective factor for the older suffering from psychological abuse. It can help family members communicate and understand each other more effectively (9, 25). Children are aware of their parents’ physical state and accept their needs. They will not show boredom or scolding, lowering the risk of psychological abuse. In contrast, family dysfunction may raise the likelihood of psychological abuse among older people. To avoid increasing a child’s mental burden, older people frequently fail to express their true thoughts and suppress their dissatisfaction, resulting in restricted family communication (40). In the long run, the intimacy between family members weakens and family function is threatened, which leads to more negative feelings in the older, such as anxiety and depression, which are more likely to cause psychological abuse (41, 42). It is clear that enabling older to actively express their thoughts to family members can help prevent negative situations from occurring in the future. Research has indicated that the use of a structural family therapy model (43) can improve family structure and member interactions, foster emotional exchange and intergenerational support, and lessen communication barriers between older people and caregivers. Subsequent studies are necessary to determine whether structural family therapy models can reduce the likelihood of psychological abuse.

The binary logistic regression analysis indicated that the factors affecting psychological abuse among home-dwelling older people were residence, monthly income, and whether had chronic diseases (*p* < 0.05). Compared with the urban, older people in rural areas are more likely to suffer from psychological abuse (OR = 3.487, 95% CI = 2.121–5.732). This is similar to the results of Oluoha et al. (44). This could be attributed to disparities in pension and family economic conditions between the rural and urban older people. Rural areas are considered fragile regions of elder abuse because, in comparison to urban areas, rural areas are geographically isolated, inadequate access to services, low socioeconomic and educational levels (42). Currently, China’s general level of social security is poor, and aged care resources in rural areas are inadequate when compared to urban areas. For this reason, older people in rural areas are more prone to experience psychological abuse. Therefore, it is vital to boost social security for older people in rural areas, as well as improve their medical conditions and living standards.

Compared with those with a monthly income of ≤ 800 yuan, older people with a monthly income of ≥ 3,501 yuan were less likely to experience psychological abuse (OR = 0.342, 95% CI = 0.119–0.987). The primary cause of elder abuse, according to Du and Chen (45), was an economic status. A good economic status can improve the health of older people, lowering the prevalence of elder abuse. On the contrary, when older people have a low monthly income, their daily living and medical treatment are also low, which has a direct impact on their quality of life and physical health, increasing the likelihood of psychological abuse. In China, the pension system needs to be reformed, and it is insufficient for older people to rely solely on subsidies. As a result, the higher monthly income of older people has a significant impact on their quality of life in later life and, to some extent, avoids the occurrence of psychological abuse.

Older people with chronic diseases were more likely to experience psychological abuse than those without chronic diseases (OR = 2.202, 95% CI = 1.356–3.576). A study of elder abuse in an Ethiopian community discovered that having multiple chronic diseases was one of the risk factors for elder abuse (46). Filipska’s research (47) revealed that chronically diseased people experience violence and abuse more often than healthy older people. This could be because, on the one hand, treating chronic diseases in older people frequently necessitates high medical costs (48), which puts further financial strain on the family. On the other hand, older people with chronic diseases are more likely to lose their ability to care for themselves and require more assistance from their spouse or younger relatives in everyday life, which increases the care burden. Pinyopornpanish et al. (49) found that caregiver outcomes like despair and burden were linked to elder abuse. Additionally, when a spouse or young relative must be taken care of an older person with chronic disease for an extended period, their natural rhythm of life is disrupted, and unpleasant emotions and psychological stress also arise. As a result, it is simple to get family caregivers to mistreat, criticize, or complain about older people, which raises the possibility of psychological abuse. In the future, how to avoid and intervene in the psychological abuse of older people with chronic diseases is worth investigating.

This study had some limitations. The primary methodological limitation is the cross-sectional survey, which makes it impossible to establish a causal link between identified correlates and psychological abuse. Longitudinal research should be conducted in the future to address this limitation. Secondly, the survey area of this study is Shijiazhuang in Hebei Province, Jining in Shandong Province, and Dalian in Liaoning Province, which excludes the economically developed city in Northern China. Future studies should include more cities and more possible factors.

## Conclusion

In conclusion, this study found a low level of psychological abuse in the survey of 465 older adults. The lower levels of PASA, IADL, and higher FACES levels were associated with fewer experiences of psychological abuse, highlighting their important roles as influencing factors of psychological abuse. In addition, psychological abuse was significantly associated with residence, monthly income, chronic diseases, and FACES. Henceforth, community managers should pay attention to the psychological abuse problem among home-dwelling older adults and appropriate interventions to monitor and avoid it. On the one hand, we should improve monitoring and early warning systems for vulnerable older adults, allowing psychological abuse to be identified and addressed promptly. On the other hand, various services like legal assistance, psychiatric counseling, family relationship mediation, and temporary refuge have undergone constant improvement.

## Data Availability

The original contributions presented in this study are included in this article/supplementary material, further inquiries can be directed to the corresponding author.
